# Transactions of the King's and Queen's College of Physicians in Ireland. Vol. IV

**Published:** 1824-09-01

**Authors:** 


*Transactions of the Association of Fellows and Licentiates of the King and Queen's College of Physicians in Ireland.*— Vol. fourth. 8vo. pp. 452, plates. Dublin and London, April, 1824.

These Transactions took a higher stand from the very first than their cotemporaries of the British Isles, and every candid examiner must acknowledge, that they continue to maintain the vantage ground, and decidedly to cast in the shade every series of transactions, emanating from similar associations East of the Irish Channel. Superiority in numbers, whether in war, literature, or science, does not necessarily ensure victory. When we compare the Association of Members in Dublin, with the "Members of the Medico-chirurgical Society," in London, it is a Spartan handful to a Persian Host! And when we examine the late volumes which these bodies have sent into the world—but comparisons are odious, and we shall pursue them no farther.

The work under review contains thirty-five articles. We shall deviate from the mode which we have hitherto pursued in analysing volumes of this description. We shall select for our Review department, those papers which involve or bear upon general principles, and, with a few exceptions, consign the analyses of insulated facts or cases to our Periscope, where they will fall in, according to the nature of the subject, under their appropriate heads.

### Art. I.

*Observations on a Species of Premature Labour to which Pregnant Women are not unfrequently liable.* By an experienced Physician.

The experienced physician should have affixed his name to this communication. Doubtless it is known to the association, or at least to the member (Dr. Brooke) who introduced the paper. We do not make this remark, with the view of detracting from the merit of anonymous writings. There are some species of literature which are properly anonymous—as reviews—and no other plan ever did or ever can succeed in that line. The late Mr. Cumberland tried nominal criticism, and it failed, although many of the articles were of sterling merit, and would

---

similar passage from the Edinburgh Review. We know not whether we are included in, or excluded from, the very few exceptions which this censorious critic admits; but in whatever class, we cannot help considering the judgment as infinitely too severe.—*Rev.*

have excited great attention had they been anonymous. But in the transactions of a society, where matters of fact and experience are to be recorded, the name of the writer adds weight to the communication, and the concealment of it generally raises doubts in the mind of the reader.

The species of miscarriage, under consideration, is as distressing to the patient as it is perplexing to the physician. Having consulted men and books respecting it, without much satisfactory information, our author wishes to excite the attention, and if possible, collect the observations of others in regard to it, through the medium of the press. The species of miscarriage is this :—

"A lady, apparently healthy, conceives and carries her child in the usual way, till about the seventh or eighth month of pregnancy, she by degrees ceases to perceive the motions of her child; and in about *ten* days or a *fortnight after* this event, she falls in labour, and a fœtus, evidently dead for some time, is expelled. This often happens three, four, five, or six times in succession, or perhaps more frequently, to the same patient, and about the same period of pregnancy. The first time such accident happens, there has generally been some cause to weaken the patient, during gestation; but, in the subsequent instances, it rarely happens that any adequate cause can be assigned. Women who have borne many healthy children have sometimes fallen into this pernicious habit, and continued it for a length of time, and afterwards had *living* children. A memorable instance of this kind occurred in the lady of a Viceroy in Ireland, about thirty years ago. In such cases, it is evident that miscarriage happens in consequence of the fœtus dying in utero." 28.

Our author asks, what are the most likely means of preventing the death of the fœtus in utero? Is it not reasonable to suppose, that the existence of the child must intimately depend on the *quantity or quality* of the fluids supplied by her?

In some cases, he observes, we have reason to think that more blood circulates in the mother's system than is consistent with the health of the fœtus—but more frequently the reverse is the case. In not a few cases he has been led to suspect *acrimony* in the fluids of the mother. "By the imprudence of husbands a venereal taint has been sometimes acquired, which required the use of mercury, and which perhaps has been insufficiently employed. The wives of such are particularly liable to the disease in question, although no unequivocal venereal symptoms shall exist." "The most likely method then, of preventing the death of the fœtus in utero, is to consider whether in the mother's constitution, there be symptoms of *redundant*, *deficient*, or *acrimonious* blood." The symptoms of the first two states need not be alluded to, as they are well known.

"I have only to remark under the second head, that, in some such cases, I have had reason to think very small bleedings, at distant intervals, of use, although little indicated by symptoms. Was this by creating a tendency to plethora? This is an effect of which venesection has been accused. Symptoms of acrimony in the fluids are more equivocal and uncertain, as well as the means of correcting it.

"In the *unimpregnated* state, sulphureous mineral waters, goat's whey in the proper season, tepid bathing, strong decoctions of sarsaparilla, and slight mercurial courses, may be tried. Where there are no decided syphilitic symptoms on *either* parent, and a healthy child in existence, and assurances of no subsequent exposure to recent infection, it appears rather unreasonable to press the use of mercury to any extent, and indeed it will seldom be submitted to." 30.

The experienced are requested to forward to the association any facts tending to illustrate this obscure and interesting subject.

In answer to these queries, a letter was read to the association from Dr. Beatty. The species of premature labour, described at the beginning of this article, early attracted Dr. Beatty's attention, and still continues to do so. As far back as 1789, when Dr. B. was resident assistant at the Dublin Lying-in-Hospital, he delivered a woman of a putrid child in the eighth month of pregnancy, and was informed, that this had been the case several times before, so that she despaired of having living issue. Suspecting a venereal taint, he proposed a course of mercury and separate beds. The proposal was complied with, and the result was a living child, in due time after the mercury had been discontinued. Several similar cases occurred to Dr. B. from that time, with similar success, but which he passes over, as resting on his individual experience. He adduces some instances, however, where he was assisted by others, as Mr. Colles, Mr. Todd, &c. in their capacity as surgeons. We shall here introduce a case or two in illustration.

"In my case book, to which I have referred, I find that in August, 1812, I attended the wife of a staymaker, who was delivered of a putrid child in the seventh or eighth month, which, she said, was the third that she had borne dead. I discovered so much of venereal affection, as to recommend that they should put themselves under the care of some experienced surgeon for the use of mercury. They applied to Mr. Colles; and when she was pregnant in the following year, Mr. Colles told me that they had not continued a sufficient time under his directions to satisfy him they were cured of the venereal complaint; which I found to be the case in July, 1813, when I delivered her again of a putrid child in the eighth month. I then declared that I never would attend her again, until Mr. Colles told me that he was satisfied with the result of the mercury used. They again returned to him, and fully attending to his directions, in October, 1814, I attended her, when she bore a living girl at the full period of gestation. She has had several living children since.

"In October, 1816, I delivered the wife of a cavalry officer of a putrid child on the eighth month. The gentleman had been on the Continent with his regiment without his wife, and had contracted a slight venereal complaint, of which his surgeon considered him well before his wife joined him in France. I could not detect any venereal symptom in the parents, but was so satisfied with the cause of the child's death, from the peculiar appearances on the body, that I recommended them to consult some eminent surgeon; and Mr. Todd was called in, who met the regimental surgeon with me, and advised the use of mercury, which was regularly persevered in by both for several weeks. After this course pregnancy was soon the result, and in November, 1817, I had the gratification of attending her when she had a living girl. She has had several living children since." 33.

Dr. B. relates several other cases of a similar nature, and adds that "he never attended any person who had dead children, and whom he suspected of venereal complaints, who did not readily submit to mercury, so strong and general is the desire for posterity," excepting one, a celebrated Courtezan, who every year produced a dead child, and would never take any means to alter this state. To the question then, "what are the most likely means to prevent the death of the fœtus in utero?" he would answer—"*the use of mercury*." It has, in every instance, succeeded with him, and he has not met with any case which he thought safe to commit to the use of mineral waters, or the other means suggested by the anonymous enquirer.

"I have," says Dr. Beatty, "met with several cases wherein very delicate women have borne dead children at the seventh month, but not putrid; and have, where I did not suspect venereal taint, constantly succeeded in avoiding the accident by a rigid confinement, even to one floor, and by a very strict attention to keep the bowels gently free, from the earliest period of gestation to the end of the eighth month; and several, to whom I gave permission to go out at that time, have thanked me, saying they were never so happy as in their confinement, and would not accept of my offered emancipation. I do not remember a single instance where good health, good looks, and a continuation of bearing living children, were not the rewards of the confinement." 36.

We think the above observations of so experienced a physician-accoucheur as Dr. Beatty, entitled to serious reflection by every one engaged in obstetric practice.

### Art. II.

### *On Tinea.* By John Crampton, M.D.

Considered as a mere local affection, occurring generally at a certain time of age, and not involving any danger to the patient's life, tinea has perhaps attracted less attention than might have been expected; and is often consigned to the care of the nurse, or nostrum-monger. It frequently, however, baffles the skill of the regular practitioner.

Our author is not aware, that there is any fixed principle or settled mode of treatment in this complaint. "Most of those," says he, "who have to prescribe for cases of tinea, adopt an experimental mode of proceeding, using various topical applications, abandoning each remedy after a short trial, and changing to others in succession equally ineffectual." On this and other accounts, Dr. Crampton is induced to offer a few observations on tinea, founded on the experience of twenty-eight cases, where he had a fair opportunity of seeing most of the forms of the disorder, and appreciating the different modes of treatment usually adopted. The patients were children from the Bedford Asylum;—they were all cured in the space of six months, some of them in a period much within that time. He does not pretend to say that none of these children had any relapse—but these relapses were very slight, and of short continuance.

There was considerable variety in the appearances of the heads of these children, according to the duration of the complaint, and the constitution of the individual; and there might be some foundation, he observes, for dividing it into the several species which Sauvages, Willan, Bateman, and Alibert have attempted to establish. Our author began by having the scalp shaved, and then the crusts or scales removed by soap and water, with poultices. After this, various external applications, such as are recommended by the different writers, were used, and they all failed—or at all events, gave but temporary relief.

"Having exhausted my patience with trials of the different topicals enumerated, as well as others needless to mention, I had by this time an opportunity of witnessing the effects of constitutional treatment in some of the patients, who require it for morbid affections independent of Tinea. Some of the children had feverish attacks, where purgatives were repeatedly given, and, in some instances, venesection was resorted to: the amended appearance of the local complaint, under this discipline employed to subdue fever, soon encouraged me to give aperient medicines every second or third day to all the children. Saline purges were preferred; their influence, after making comparative essays of different cathartics, appeared to be most decisive.

"It was further observed, that the scrophulous children with large bellies, and other descriptions of patients where rhubarb and other tonic purgatives were exhibited, in addition to an amended state of general health, seemed to improve, as to the local disease, with more celerity than otherwise healthy children, where topicals only were employed.

"Another expedient which had been adopted also, with views originally unconnected with the treatment of Tinea, I found showed a considerable share of power in advancing the cure. Warm baths had been directed for several inmates of these wards, when the skin was observed to be harsh and dirty, also for those who were convalescent from feverish attacks; in addition to an ameliorated state of the skin in general, and of the cutaneous secretion, the appearance of the scalp was palpably and materially improved in those who took these baths.

"I had thus, after some time, learned from experience to appreciate the advantage of constitutional treatment, and to trust less to topicals; I discarded therefore the variety hitherto in use, and confined myself chiefly to simple poultices, aided by a constant use of purgatives and the tepid bath. A certain number of the children were bathed every evening in turn, so that each of them had a bath every third or fourth evening.

"The treatment which I finally adopted was, first to use poultices, giving cathartics every second morning, and the bath every third evening; during the time the poultices were discontinued, the liniment with lime-water and oil was applied." 60.

When the common oatmeal poultice was not sufficiently strong to remove the crusts, one was constructed of soap, reduced to a stiff jelly. This poultice was productive of rapid amendment. It very quickly dissolved and removed all hardened, lymphy, and other morbid secretions, though it was considered by the patients as a very drawing and rather severe application, occasioning some irritation and smarting. This, however, as well as every other external means, failed eventually, unless accompanied by the internal or constitutional treatment abovementioned.

For Dr. Crampton's commentaries on the sentiments of Alibert, Bateman, Plumbe, and other dermological writers, we must refer to the volume itself.

### ART. III.

### *Case of fatal Result from Mercurial Ointment.* By J. CRAMPTON, M.D.

We fear that the mystery which hangs about the pecularities of constitution giving rise to mercurial diseases, will not be unveiled by Dr. Crampton's present paper. That those who have made *post mortem* examinations, in such cases, may have neglected to ascertain the state of the mucous membrane of the intestinal canal is highly probable, for it is very true, as Dr. C. observes, that, " until within the last twenty years," (he might have brought the period much lower, and applied the reproach to very many of the present day) " in the examination of bodies after death, anatomists rarely slit open the intestines to examine their inner membranes, satisfied if on their exterior they presented the usual healthy appearances."

Much has been written by Toxicologists and others respecting the effects which the oxydes of quicksilver produce on the stomach and bowels, when given internally in an over-dose; but our author has not been able to find any observation as to the lesions of texture induced by the external application of mercury, in the form of ointment, except where patients have fallen victims to its severe effects on the mouth and fauces. It is on this account he has been induced to record the following case, more especially " as the light which dissection throws on it may (he thinks) enable us, by an appropriate treatment, to save patients, who might otherwise fall victims to the same fatal train of symptoms." We shall considerably abridge the case.

J. Jones, æt. 20, naturally robust, was admitted into the Whitworth Chronic Hospital on the 7th November, 1821, for ascites and general anasarca. Cathartics and diuretics having failed, he was ordered, on the 30th of November, to rub in half a drachm, twice a day, of mercurial ointment on the abdomen. When three drachms of the ointment were used the mouth became sore, and the frictions were discontinued. He was then directed gentle aperients daily. On the 8th December the parotid and submaxillary glands, together with the whole integuments of the face, became much swollen, the secretion of saliva being still copious. Twelve leeches were applied to the tumid gland, which afforded some relief. On the 10th, the pulse being full and frequent, blood was taken from the arm, and two days afterwards 15 leeches applied to the tumid parts.—14th, the breathing was alarmingly laborious, and 15 ounces of blood were abstracted from the arm. On the 15th and 16th, blood was discharged with the saliva, and there was every appearance of amendment; but on the night of the 16th, the salivary discharge ceased—the swollen countenance subsided—the tumour of the glands and the swellings of the abdomen disappeared. The patient lay in a state of stupor, as if labouring under an apoplectic fit, his breathing stertorous, pupils insensible to light, powers of speech and motion abolished. " Apparently there was every reason to suppose that effusion had taken place, either into the ventricles or between the meninges of the brain." He died on the night of the seventeenth.

*Dissection.* No anasarca—no prominence of the abdomen. Dura mater slightly opake and thickened—no preternatural turgescence of the blood-vessels or effusion into the ventricles. Thoracic viscera were healthy. In the abdomen five or six ounces of fluid—liver enlarged and diseased—mucous membrane of the small intestines, more especially of the ileum, of a bluish green colour, ulcerated in many parts, " or rather appearing as if corroded by some active chemical substance."

" On reviewing the appearances after death, and comparing them with the symptoms, the first remark which occurs is the uncertainty of medical prognosis. Antecedent to death, almost any physician of experience would have pronounced the disease apoplexy of the metastatic kind; that serous effusion took place suddenly on the disappearance of dropsy from other cavities; or that rupture and extravasation of blood occurred from the new determination excited by the mercurial irritation. Of such occurrences in dropsical diseases I have seen several instances, where the appearances just described, of effusion, &c. were found in the brain on examination after death, Such sudden transitions have, according to my experience, happened oftener where mercury was early employed, than under other modes of treatment. I have not observed such sudden and such fatal metastases since I have been in the habit of using the lancet, and other modes of sanguineous depletion, in recent cases of dropsy: after the removal of inflammatory symptoms, mercury here, as in other diseases, is safer and more likely to atchieve what the practitioner may desire." 99.

A careful review of this case does not lead us so straight forward to Dr. C's. conclusions, as he may expect. We confess we cannot clearly see how this case shews us, " what particular texture is affected, where mercury, *by inunction*, proves injurious, and how death takes place." The ulceration in the mucous membrane of the intestines is singled out by Dr. Crampton from among other lesions, and pronounced, without a shadow of proof, as the effect of three days mercurial inunction, while a diseased liver, and thickened dura mater are matters quite unworthy of notice. Has Dr. Crampton never seen ulceration of the mucous membrane take place when no mercury was rubbed on the surface? What becomes of Dr. Broussais's researches, in which this state is shewn to obtain in almost all febrile affections, whether mercury may have been administered or not?—In fine, we venture to differ from our able and respected author, not only as to the ulceration being the effect of mercurial friction, but as to its being the cause of death. The very day before dissolution Dr. Crampton thought the patient would recover—but suddenly a state resembling apoplexy supervenes, and the man dies. Is this like death from ulceration of the bowels? The man died, in our humble opinion, from cerebral affection; and if Dr. Crampton expects to find organic and visible changes in the brain, in every case where death results from its physiological lesions, he will be greatly disappointed. We have no objection to the following therapeutical observations.

"As to the mode of prevention, or the method of treating diseases arising from mercury, an observation or two will suffice: indeed, there is less hazard now of meeting with such occurrences, as patients are generally prepared, previous to a mercurial course, by purging, by low diet, by the antiphlogistic regimen, and, in some instances, by detractions of blood. The mercury is not thrown in at once, as was formerly the expression and mode of practice, at a time when the constitution was perhaps already too much excited from other causes, and when the remedy was taken much to the disadvantage of the patient. If these precautions are observed, there will be less chance of any unpleasant symptoms arising from the use of the remedy, than if it is adopted with more precipitancy.

"When symptoms, such as are described in the case which makes the subject of this paper, are met with, a treatment suited to prevent and remove intestinal inflammation should at once be adopted. Remedies also to counteract the disordered state of the cerebral functions should be prescribed. On either of these points of practice I feel it unnecessary at present to expatiate." 107.

### Art. IV.

### *Case of Chorea.* By John Crampton, M.D.

Our author, who appears to be a liberal contributor to this volume, observes that little light has been thrown on chorea by anatomical investigation. Practical writers have, however, endeavoured to supply the deficiency, and experience has furnished us with many remedies for this curious disorder. The following case is remarkable as occurring in an adult—and at a period of life when such a disease is little to be expected The instance, however, is not solitary, as Dr. Maton has related a case of chorea occurring in a woman 70 years of age.

*Case.* Anne M'Kelvey, Ætat. 42, unmarried, was admitted into the Whitworth Hospital, 12th February, 1821, labouring under a severe paroxysm of chorea, which had lasted five days. The hands and legs, sometimes one, sometimes all, are affected with a hurried convulsive motion, appearing to the spectator as if the patient were beating time to a musical instrument. She speaks in a hurried manner, and expresses great uneasiness when any of the limbs affected are held by another person. The bowels are constipated, tongue looks unhealthy, pulse weak and slow, no catamenia the last two years. She had had uninterrupted health previous to September 1819, at which time she became affected with hysteria, and continued subject to it until about twelve months ago. It was four months since the chorea first appeared. She was directed to have a fetid enema, and afterwards to have every night some rhubarb, hyoscyamus, blue pill, and colocynth to act on the bowels. She was also directed to take a quarter of a grain of the argentum nitratum thrice a day. This last was gradually increased to two grains three times a day, until the 20th March, when it was omitted, the chorea having entirely disappeared.

The pathology of chorea is unsettled, and so is the treatment. The following remark is all we shall extract from Dr. Crampton's observations on this complaint.

"Although certain general principles should guide our practice, yet it would appear that no settled plan, no specific remedies, can apply generally to all cases of Chorea we may meet with. Our measures must be varied according to the particular organ or system which seems to suffer most: at one time our views must be directed to the vascular system in the head, also to the state of the liver and digestive organs; again, the condition of the nervous system chiefly claims attention. In other instances a combined treatment must be adopted, using at the same time both evacuants and nervous medicines." 121.

### ART. V.

*Cases of Hydrocephalic fever.* By HENRY BALDWIN EVANSON, M.B. R.I.A.

The two first cases died in a day or two after they came under the care of the reporter, and on dissection presented the usual appearances. The third case was that of a boy, ten years of age, who was found by our author lying in a state of stupor, apparently in the second stage of the disorder. When spoken earnestly to, he replies distinctly—raises his eyes with difficulty—pupils dilated, but moveable—tongue very foul and yellow—pulse not much accelerated—general uneasiness, but no distinct pain—slight vomiting.

"Had this been a solitary case it might perhaps have been mistaken for fever; but the dilated pupils, the dark green, ponderous, and slimy stools, and the previous history, sufficiently designate the disease. Leeches, vinegar and water externally, calomel, and the common senna mixture, were immediately brought into use, but the next day he was much worse; coma had increased, and his tongue and nostrils were covered with a crust as black as soot. Leeches were again applied, and he was ordered *submur. hyd.* gr. iii. *pulv. Jac. veri*, gr. iss. every fourth hour, the purging mixture being continued as before." 158.

Profuse bleeding took place from the leech-bites, one of the branches of the temporal artery having been cut. A blister was applied to the neck, and a discharge kept up by savin cerate. There was a manifest improvement after this, and by persevering with the powders, the crust soon came away from his tongue and nostrils. This improvement, however, was but temporary, as the alarming symptoms soon recurred—his speech thick and faltering, like that of an apoplectic—vision appearing double. The acetum colchici was now given, with camphor mixture.

"When he had taken two-thirds of this, the double vision was gone, and his bowels and kidneys were acted on so powerfully, that it was deemed right to omit the mixture until next visit. But we had no reason to congratulate ourselves on doing so, for he fell back again, and we found it necessary to put him under its influence as quickly as possible, at an increased dose. This mixture was subsequently continued at varied intervals, of double strength; and lastly, its diuretic power was increased by adding acet. potassæ, ʒii. The quantity of urine it produced *per diem* was generally about ℔ iv. It sometimes sickened the patient's stomach; and latterly there was a good deal of pain about the neck of the bladder." 159.

When the third stage declared itself, they gave opium—fifteen drops of the tincture in camphor mixture every night. "This was found most effectual after copious depletion by leeches." These were applied to the temples twelve times in the course of the disorder, from eight to ten at a time, but always with advantage. A high degree of delirium sometimes remained after the blood ceased to flow, but the opiate then took effect. An eruption was exuded on each tibia by the tartar emetic ointment. The above three cases were three members of the same family.

"In proposing a new remedy, I feel anxious to have it understood, that I look upon it merely as an additional weapon, which will not supersede any of those wielded hitherto, except digitalis, and that not in all cases. Of those in use, blood-letting, purgatives, and mercurials, hold the first rank. The first of these is the basis of all the rest, yet it is not employed with freedom amongst medical men *at large*. Some withhold the lancet from timidity, and others trust too much to purgatives, &c. The debility attending this disorder is not such as we see in typhus, in which, notwithstanding the state of the blood-vessels, we find it highly beneficial to draw off a moderate quantity of their contents by one or more abstraction of blood, either local or general; surely then we should avail ourselves of the lancet with boldness in every stage of this disease, except in the stage of collapse, as the termination of the disorder is proved to be the consequence of increased action in one set of vessels, or of congestion in the other: both these states most probably exist in concurrence. I see nothing to forbid general blood-letting in the third stage but a feeble or thready pulse, connected with a sinking and exhausted state: mere local depletion seems sufficiently active only for very young children." 164.

The chief efficacy of the colchicum (if it possess any) consists, thinks our author, in its power of promoting secretion.

For Dr. Evanson's criticisms on Dr. Golis's treatise, we must refer to the work itself. We fear Dr. E. is very inadequate to the task of commentator on such a man as Golis.

### ART. VI.

*A Letter on Variola after Vaccination.* By JOSEPH CLARKE, M.D.

Much of the alarm, Dr. Clarke thinks, respecting the security of vaccination, has arisen from want of due attention to a few words of Dr. Cullen's definition of variola—" Papulæ phlegmonodeæ, quæ spatio *octodierum* in *suppurationem* abeunt."

" Can it be deemed unreasonable to ask practitioners to keep these words constantly in mind, and to wait with patience till the eighth day after the eruption. If pus can *then* be found in the pustules, the existence of variola can no longer be doubted; but until this period arrives, no prudent man should venture positively to decide. Let it be remembered that varicella is defined by the same high authority—" Pustulæ, variolæ similes, vix in suppurationem euntes, et post paucos dies desinentes." 182.

In several recent cases, variola and varicella, says Dr. C. have been confounded by practitioners of experience and ability. " These errors must have arisen from opinions formed before the *duration or contents* of the pustules could be ascertained." The purport of the following remark is to guard his brethren against hasty and erroneous judgments, which unnecessarily disturb the peace of families, and tend to throw discredit on medical science.

" During the last few years I have seen many cases of spurious eruption, very like, in all symptoms, to severe small-pox, for the first eight, nine, or ten days, from sickening. On the sixth day of the eruption, or at latest on the seventh, the pustules declined rapidly, the fever subsided, and on the eighth day not a vestige of pus was discoverable. Had these been cases of genuine variola, the patients, instead of amendment on the sixth day of eruption, would have remained in a state of increasing danger for many days, and would have experienced a slow recovery." 183.

At the same time Dr. Clarke does not contend for the absolute infallibility of vaccination. Although failures must be expected to multiply as the practice gets into the hands of ignorant men, yet it is satisfactory to learn that, " since the beginning of this century, when vaccination was introduced generally among the upper ranks in Dublin, no family has lost a child in previous good health, by small pox after vaccination —nor has even one eye been extinguished by this pestilential disease."

Two of the worst cases of secondary small pox which our author has met, occurred in mothers who had been, in early life, inoculated with small pox virus, and considered as secure against its future influence. Both had the disease severely—one dangerously. The following sentiments from a man of Dr. Clarke's experience deserve record here.

" These facts justify us in concluding that neither inoculation for small-pox, nor vaccination, afford perfect security ; but that vaccination, conducted with judgment, protects the human frame against the dangers of small pox, I entertain no doubt. If the general principle of vaccination affording protection were unsound, it is not *tens* of failures we should now have to record, but *hundreds*." 184.

A report from the Cow-pock Institution closes this paper. It does not differ materially from other reports of similar establishments. It admits the pretty frequent occurrence of *varioloid* disease after vaccination, but contends that, in no case, did it prove fatal.

### Art. VII.

*Case of a young Woman who has discharged, and continues to discharge from her Stomach a number of Insects, &c.* By William Pickells, M.B. one of the Physicians to the Cork Dispensary, &c.

This is a very curious case, and may afford matter of speculation for the " equivocal generation" and "omnia ex ovo" philosophers. The wretched subject of the paper is Mary Riordan, aged 28 years, and who is now weakened by long suffering, but still exhibiting traces of an originally robust form and constitution. She is of highly nervous sensibility, melancholic temperament, and religious frame of mind. Her melancholy mood may be traced to the death of her mother, over whose grave she daily poured the tribute of filial sorrow for months, regardless of the rigor of the season. On one of these occasions, overcome by the poignancy of her feelings and fatigue, she was found the following morning lying over the grave, in a state of insensibility, having spent there the entire of a winter's night.* During the last six years, she has laboured under hæmatemesis, and occasional convulsions. Our author first saw her in July 1819, under fever, for which she was removed to the House of Recovery, Cork. Here she was found to have hepatic affection, accompanied by dropsical swellings and hæmatemesis. Under the latter complaints, she laboured for nearly the next 18 months, when she again came under Dr. Pickells' cognizance. In the above-mentioned interval, she became affected with retention of urine, requiring the use of the catheter ever since. "On some occasions, when the urine was suffered to accumulate, she was relieved from the distention in the hypogastrium by urinous vomiting." This is another fact in favour of one side of the controversy carried on a few years ago, respecting the possibility of urinous vomiting. The patient also laboured under a disease resembling catalepsy, attacking her suddenly, and fixing her limbs in whatever posture they happened to be at the time of seizure. In the intervals of these attacks, she complained of various symptoms, as vertigo, head-ache, tinnitus aurium, perversion of the senses of sight, taste, and smell. At one time she had complete amaurosis for a fortnight, which appeared to be removed by an ichorous discharge from the ears.

"The hæmatemesis during this period continued to occur more or less frequently, the quantity of blood thrown up varying, on an average, from a pint to a pint and half. She used to complain of a peculiar gnawing pain about the pit of the stomach; and the irritability of the abdomen was at times so great, that the slightest touch, or even the wind of a person passing by her, was sufficient to induce convulsions." 192.

Her appetite was variable, and she frequently vomited up her food. She had been in the daily habit of eating large lumps of chalk, having found it to mitigate the burning sensation in her stomach. Her mouth was constantly parched, and her thirst

* It is well known that the Scotch and Irish exhibit far stronger proofs of filial and parental affection than the English. Much of this difference is attributed, and we think justly so, to the operation of the poor laws, which certainly tend to sever the ties of nature, so strongly knit between parent and progeny throughout the human and every other animal race. Yet we cannot think that the alledged cause is entirely equal to the assigned effect. Allowing that man is the creature of surrounding circumstances, we know that these circumstances, when they have long operated, produce an *hereditary disposition*, which continues to exist long after the causes from which it originally sprung have disappeared. Something of this kind, we imagine, must be taken into account, in aid of the baleful influence of our pauper institutions, and in explanation of the marked difference between the Irish and Scotch peasantry on one side, and the English on the other.—Rev.

excessive. An intolerable fœtor issued from her throat—tongue constantly white, and bowels obstinately costive. Catamenia almost gone since the commencement of the discharge of insects; for three years previously to which she had been often at the verge of the grave, and the last rites of the church had been administered to her not less than fourteen or fifteen times.

The discharge of insects commenced on the 3d of April (1822, we believe, for great neglect often takes place in not putting down the year in reports of this kind), and our author visited the patient almost daily, between that period and the 29th of April, 1823. The first eruption of insects (larvæ) took place after a violent mental emotion, and was immediately preceded by a discharge of blood from mouth, nose, and ears. We cannot attempt to describe the various insects, in all stages of larva, pupa, and perfect animal, minutely detailed by Dr. Pickells, and represented in the plates. The curious must have recourse to the volume itself. But we may observe that Dr. Pickells and other medical gentlemen were frequently witnesses of the ejection of these animals, and there appears no reason whatever to suspect the slightest disposition to imposture on the part of the patient.

"Of the larvæ of the beetle, *I am sure I considerably underrate* when I say that, independently of above a hundred evacuated *per anum*, not less than *seven hundred* have been thrown up from the stomach at different times since the commencement of my attendance. My own reckoning, during my personal attendance, gives upward of four hundred; but in this calculation is not included the number thrown up during my absence of three months, a period marked by the expulsion from the stomach of such larvæ, almost daily, in some instances, as reported, to the amount of above thirty at a time. A great proportion were destroyed, from an anxiety to evade publicity. Many too escaped immediately after having been vomited, by extricating themselves quickly from the vessel, and running into holes in the floor.*

"Upwards of ninety were submitted to Dr. Thompson's examination, nearly all of which, including two of the specimens of tenebrio molitor, I saw myself thrown up at different times. The average size was about an inch: many, however, which I measured, were an inch and a half in length, and four lines and a half in girth.

"The larvæ of the dipterous insect, though voided only about seven or eight times, according to her account, came up almost literally in myriads. They were alive and moving. None of those have been known to have been discharged within the last seven months.

"* It may seem ridiculous, but such is the fact, that the females who attended during the operation of an emetic, sometimes carried their apprehensions so far as to secure their petticoats below."

"The larvæ of the beetle were, with few exceptions, lively and vigorous in the extreme; nor was it possible, without a feeling of horror, to view them frisking along the bottom of the vessel in which they were preserved, occasionally expanding their jaws, and extending their denticulated feet, or 'talons,' as their unfortunate victim used to call them. Some, which were apparently dead, revived upon exposure to heat.

"Enclosed in empty pill-boxes several lived upwards of a month. Mr. Clear, of this city, has succeeded in preserving some of the earliest thrown up, still alive, now after an interval of a year, by keeping them in little pots filled with clay, and so secured as not to exclude the air. Some specimens of the larvæ of blaps, which I gave to Mr. Clear, when kept in flour, were observed to be continually running to the surface, as if impatient of their situation, and seemed not to thrive; but when placed in clay quickly buried themselves, and seemed to enjoy their native element." 209.

Dr. Pickells was naturally anxious to ascertain, if possible, the mode of introduction of the insects—or rather of their ova. The patient's answer to one of his questions unfolds a tale which forms a degrading instance of superstition in the nineteenth century.

"When she was about fifteen years of age, it appears that two much-respected clergymen of her persuasion having died, she was told by some old women, that if she would drink daily, during a certain period of time, a portion of water imbued with clay, taken from the graves of these clergymen, she would be secured for ever against both disease and sin. She accordingly walked to Kinsale, a distance of twelve miles, where one of the clergymen was interred, and succeeded in bringing away an apron and pocket-handkerchief full of clay from his grave. To this she added, upon her return, a handkerchief and some mugs full of clay, obtained from the grave of the other clergyman, who was buried in this city. Her practice was, to infuse water from time to time, according to the exigency, in a vessel containing a proportion of clay so collected, the mixture having been always allowed to rest until the grosser particles of clay fell to the bottom." 212.

The blaps mortisaga is well known to inhabit church-yards and similar situations; but the occurrence abovementioned having happened twelve years prior to the first discharge of the insects, it seems difficult to reconcile with analogy the supposition of their having so long remained in the system. It is not improbable, however, that these insects, in various stages of development, may have been discharged long before the time they were first recognized. It appears indeed that, upon minute enquiry, it was found that, four years prior to the date stated above, the patient had voided *per anum*, in consequence of a purgative, a few of what appear to have been the larvæ

of the beetle, since become so familiar to her. To our author it seems more probable that the insects were taken into the mouth, during the night which this infatuated woman passed in the church-yard, about eight years ago.

We have gone farther into the details of this case than we otherwise should have done, from a conviction that the irritation of living animals, (the vermes we mean,) in the primæ viæ, is productive of far more morbid phenomena than is generally supposed. We have seen hydrocephalus, epilepsy, and many grave diseases *irritated* by worms in the bowels, and we believe that many cases of supposed cure of these disorders were only destruction of the parasitic animals that gave origin to phenomena resembling them.

### Art. VIII.

*Two Cases of Successful Removal of Tumours from the Neck; with Observations.* By R. Adams, A.B. &c.

These were very formidable operations indeed, and do great credit to the skill and intrepidity of the operator. The first case evinces the practicability of the safe excision of a tumour which projected externally from the side of the face and neck, while it passed inward to the base of the skull, "completely occupying the natural situation of the parotid gland." In the second case the tumour was remarkable for its size and extensive attachments, being larger than the patient's head, who was an aged and debilitated female. The result of this case Mr. A. observes, bears on a subject upon which the opinion of the surgical world is at variance—some questioning the propriety of undertaking any operation where the disease is so extensive, while others, admitting its expediency, think it unsafe to operate without previously securing the common carotid.

"Reasoning from these cases, I shall endeavour to show that this last is a measure which can contribute but little, if at all, to the safety of the operation, while it must considerably diminish the subsequent chances of the patient's recovery." 224.

*Case* 1. B. C. ætat. 34, a thin delicate woman, came to Dublin with a large tumour, of an oval form, obliquely situated on the left side of the face and neck.

"From its greatest height, which corresponds to a line drawn from the eye-brow to the summit of the cartilage of the ear, it extends downwards to within two inches of the sternal articulation of the clavicle; anteriorly it reaches to within one inch of the angle of the mouth, and passes posteriorly the mastoid process, corresponding for some extent to the anterior edge of the trapezius. The circumference of the neck of the tumour amounts to fifteen inches and an half, and the largest part does not exceed this measurement more than one inch; so that the tumour does not hang pendulous on the neck, but firmly stands out at the distance of five inches from the parts which afford it attachment. It is rough and tuberculated on its exterior surface, and of a very firm consistence, preventing the complete opening of the mouth. No part has, apparently, been displaced, except the inferior lobe of the ear, which the tumour, in its progress upwards, has carried along with it, so as completely to intercept the entrance of air into the external meatus." 225.

The origin of the disease was traced twenty years back, when a small swelling was perceived under the ear, at first moveable but afterwards fixed, and gradually increasing ever since. The pain, which is seldom felt except in spring and autumn, is inconsiderable; but, the deformity rendering her an object of curiosity, she came to the metropolis for the purpose of undergoing an operation.

"Upon closely examining the tumour, I found, it is true, that it appeared somewhat fixed in the situation where it covered the parotid; but that all that portion which lay upon the side of the neck was in some measure moveable, and could be lifted off from the side of the larynx and mastoid muscle: and although it sunk too deep beneath the ear and angle of the jaw to permit the fingers to pass or ascertain its connexions here, and at the same time prevented the complete opening of the mouth, still, on the other hand, deglutition was but little impeded; and on due consideration, and a careful inspection of the fauces, I became convinced that the pharynx was not as yet too deeply concerned: I felt no hesitation therefore in recommending her to submit to the immediate removal of the disease, to which she gladly assented.

"May 16th, 1818. With the assistance of Messrs. Colles, Wilmot, Duggan, Cusack, Harrison, and in the presence of other friends, I proceeded this morning to the excision of the disease in the following manner: the patient placed horizontally on a table, her head properly disposed to the light, I commenced by making two incisions, extending from the highest to the lowest part of the tumour, comprehending between these an elliptical portion of skin left attached to its surface. I next dissected the skin from its anterior part, turned it towards the cheek, and separated the cyst to some depth.

"Difficulties now occurring in the removal of the tumour, where it passed behind the ascending ramus of the jaw, we endeavoured to raise it from behind, and but partially succeeded, until by firmly grasping and pulling it out from the neck, we had cut those parts of the capsule which, connecting it to the subjacent parts, felt most resisting. Having raised it from below upwards, it remained to detach it from the parotid space, into which we found it, as it were, firmly impacted; by the cautious use, however, of the knife, the cutting edge of which was always presented towards the tumour, and by the exertion of some force, the whole suddenly came away. Lastly, some portions of remaining capsule, together with the anterior part of the parotid gland, somewhat altered from its natural appearance, were dissected from the masseter muscle. Numerous wounded arteries poured out their blood, but were quickly stopped by the fingers, and tied after the operation. As the parotid duct did not present itself, it must either have been pushed from its usual situation, or have degenerated from its natural appearance, of which the latter appears to me the more probable." 227.

The patient bore this formidable operation with great steadiness. It was found that the tumour had reposed on the side of the larynx, and in great part on the sterno-mastoid muscle. Above and in front, the whole of the masseter muscle was dissected clean, and behind it the ramus of the jaw, and anterior edge of the mastoid muscle was, for some extent, exposed. Between the last-mentioned parts the tumour had buried itself into a deep cavity, bounded behind by the mastoid process, before, by the back part of the articulation of the jaw and pterygoid muscle—above, by the meatus auditorius and the ear, to the root of the styloid process. In short, the space was entirely disclosed which is naturally occupied by the parotid gland, "not a vestige of which was to be seen." The weight of the tumour when removed was one pound six ounces. It exhibited a cellular structure, firm in general, but soft in some places. The whole was surrounded by an adherent capsule, which sent projections between its numerous lobes to be connected with small cysts. In its yellow colour and consistence the interior of the tumour seemed to approach to the state of carcinoma, but it wanted that fibro-cartilagenous texture which many think essential to the morbid structure. She had strong symptomatic fever on the fourth day. But by the fourteenth day she was able to walk out, and in six weeks the wound was completely healed. It is now five years since the operation, and the patient remains in perfect health.

Our author has made some surgical reflections on the above case which will be read with interest by the operator. We can only glance at a few of the topics discussed. We all know the violent disputes that have existed among surgeons, respecting the propriety or possibility of extirpating the parotid gland. This case, Mr. Adam observes, may be probably quoted both for and against such an operation. So imposing were the appearances here, that some of those present felt satisfied that the gland was actually dissected out from its deep cavity. Our author himself would have come to the same conclusion had he not been prepared for a closer examination of the subject by the doubts so strongly expressed upon the possibility of such an operation.

"Upon a little reflection I became convinced that this was one of those cases of simple encysted tumours, which, dating its commencement from some imperfectly resolved lymphatic gland developed over the parotid, had, by increasing size and pressure, effected the absorption of all that portion of the salivary gland which lay beneath it: indeed, so complete was the obliteration of this organ, that the only part of the true glandular structure which the most careful examination could recognize, was that small portion which, lying over the masseter muscle, was not covered nor compressed by the tumour." 232.

Our author is aware that this case may be looked upon in quite a different light by others, and considered available to prove that there is nothing in the relative position of this gland that should deter us from its excision. As the portio dura of the seventh pair was divided with impunity, while those vessels which were not obliterated by the pressure of the tumour were secured with little difficulty, so the internal jugular vein and carotid artery were clearly exposed and easily avoided.

"But it will require but little reflection to satisfy us that the removal of an *encysted* tumour from the neck, no matter what be its relations, can never be complicated with the difficulties naturally to be encountered in extirpating the *diseased* parotid gland; nor can the relation of such cases by any means convince us of the prudence of undertaking such an operation. Such observations may be stretched so far as to show that there is nothing in the mere locality of the *healthy* parotid which should absolutely forbid all efforts to extirpate it; but if for a moment we consider the true scirrhous state of this gland, which surgical anatomists have in view when they protest against the propriety of making any attempts to remove it, in which a carcinomatous action is supposed to have commenced in its substance, determined its enlargement, and constituted a firm, inelastic swelling, which, even in its commencement, never had been moveable, we must, I am persuaded, coincide with those authors who are against all surgical interference in such circumstances. Nor will we, I imagine, if we consult the history of such a disease, have reason to question the prudence of this resolution, as we are informed that such affections are observed to remain stationary for years, while our experience in almost all our operations for the removal of cancerous disease will induce us to place but little confidence in their efficacy." 234.

Our author finally observes that, while on the one hand he has endeavoured to shew that any attempt to extirpate the *diseased* parotid gland is, on every ground, objectionable, yet on the other, he would wish to represent the absolute necessity of removing those encysted tumours occasionally found occupying the parotid region. It is to be regretted, however, that there is, too often, much difficulty in distinguishing between these cases—and therefore the judgment of the surgeon must always decide according to the circumstances of the individual case.

*Case.* 2. Bridget Daly, 68 years of age, has been for some years incapable of making any effort to support herself, being afflicted with a growing tumour, which now covers almost the entire of her neck, overhanging the chest, and its weight requiring constant attention. The disease commenced 30 years ago, beneath the angle of the jaw, as a small hard tumour, remaining stationary for the first few years, then gradually, though very slowly, increasing.

"The size which the tumour has at length acquired is very considerable, the highest part of it is situated beneath the right ear, the cartilage of which it has pushed upwards; from this its attachment extends obliquely forward over the ramus and angle of the lower jaw for two inches beyond the chin to its *left* side; posteriorly it passes the mastoid process, and descends towards the top of the shoulder on the border of the trapezius for two-thirds of its extent; there leaving the muscle, the line circumscribing the inferior part of the neck of the tumour passes downwards in a semi-circular direction over the sternal articulation of the left clavicle, which it touches, to the edge of the mastoid muscle of the opposite side, and then ascends to the lower jaw along the left side of the larynx. This circumference of the neck of the tumour measures *twenty-four inches*, and comprehends every part of importance in the cervical region; above, the tumour does not merely overlap the lower jaw, but seems to come as it were from behind and within this bone from the space above the os hyoides and floor of the mouth; from which, descending to the clavicle, it covers the whole of the anterior part of the neck, completely overlapping the larynx and trachea, which have been carried by the tumour to the left side. From this, its smallest part, although the most important for our consideration, the tumour is projected forward, and at the same time across the neck, increasing so much in size, as in the anterior view it conceals the entire of the neck and clavicle, overhanging the shoulder and thorax to the third rib, upon which however, except when the head is bowed, it does not rest, a circumstance which, when its great weight is taken into consideration, indicates some firm and bony attachment above; it is of a stony hardness, with many irregular eminences on its surface." 240.

Much doubt was entertained respecting the propriety of an operation under such circumstances. But as it was clear that, if some active means were not employed the patient must soon die, her difficulty of breathing and oppression being such that, whenever she walked her lips became livid and she was threatened with suffocation, it was determined to give this poor woman a chance for her life.

*Operation.* "Dec. 28, 1819. The patient placed on a table, with the same experienced assistants to whom I was indebted for aid on the former occasion, and many of my younger friends, I proceeded this morning to remove the disease in the following manner: commencing by an incision from the ear across the chin down to the cyst of the tumour, which was instantly followed by a profuse bleeding, and before we could proceed farther, it became necessary to tie many vessels; I then continued the dissection in the same line, endeavouring to detach the tumour from the surfaces of the lower jaw, to which, as I anticipated, I found it very firmly attached, and from this neighbourhood it derived its chief supply of blood. Nothing, indeed, could be more unpromising than this stage of the operation; every new incision was followed by a gush of blood, which would not permit us to proceed until the vessels were secured, among which the external maxillary, or facial, bled most profusely; but after some firm connecting bands, which held up the tumour, were felt, and carefully divided, it dropped a little from beneath the ear and the jaw, and the arteries which were not tied ceased to bleed. It was now time to detach it below from the neck, which was more easily accomplished; after making a circular incision, and raising up with one hand the tumour from the neck, we met with but little difficulty until we arrived at that point where it lay upon the sterno-mastoid; here the tumour was so firmly attached, that it was impossible to dissect it from the muscle, which it therefore became necessary *to split from below upwards*, to disengage the diseased mass. It now only remained to detach it from its deepest connexions beneath the ear and lower jaw. As there were no very firm bands to be divided here, I abstained further from using the edge of the knife; but sometimes with its handle, and sometimes with the fingers, but chiefly by twisting it, it at length yielded, and came away entire. As soon as the patient recovered from the faint into which she fell immediately after the operation, many small vessels were secured, and the wound as quickly as possible closed; it was necessary to unite several points of skin by suture. The poor woman bore this painful operation with much fortitude, only occasionally complaining when the larynx was disturbed by the displacing of the tumour. In the evening there was slight hæmorrhage, which was stopped by pressure, without opening the wound." 244."

Nothing particular occurred till the fifth day, except a gradual acceleration of pulse and increased heat, but not more than might be expected. On the sixth day there was a remarkable depression of strength, head-ache, nausea, quick small pulse, palpitation, slight delirium. On the seventh day she was so ill as to be reported as moribund. Calomel, opium, and digitalis were prescribed. The mouth became quickly affected with the mercury—the pulse fell—the tongue cleaned. "From this time her recovery was slow, and without interruption gradually progressive." 245.

The weight of the tumour when removed was 5 lbs. 7 oz. and although there were numerous vessels spread over its surface, a section of it exhibited no vascularity. Towards its large extremity was a cavity containing nearly a pint of an albuminous fluid. In this fluid and in the cancerous organization of the morbid mass, our author was concerned to perceive a melancholy promise of return of disease—which time has with too much truth realized. But, upon the whole, our author thinks that this poor woman has been amply repaid for her courage in submitting to the operation, by a three years' comparative state of comfort—her respiration having been rendered free, and her life having been undoubtedly prolonged. He cannot doubt, that if an earlier operation had been proposed and executed, before a cancerous action had seized upon the tumour, the result would have been as fortunate as in the preceding case.

From attentive consideration our author is convinced that the operation, however modified, of tying or throwing a ligature round the common carotid, should never be made a preparatory step to the excision of a tumour from the neck. It increases the quantum of symptomatic fever afterwards.

"But it is not merely that I object to the practice as likely to entail a dangerous consequence on the operation, which it has been supposed to render more secure for the moment, but I feel much disposed to question the utility, as well as prudence, of the proceeding, as I am of opinion it cannot prevent, and greatly doubt it can even moderate the hæmorrhage; an advantage which can, at all events, as well be derived from temporary compression of the exposed vessel, without so much endangering the life of the patient, by exposing him to the double peril of fever resulting from an extensive wound, and that necessarily succeeding the ligature of a large arterial trunk." 251.

Our author feels persuaded that the external carotid can, in most cases, be secured before the anterior part of the tumour is detached, or the great maxillary artery opened; and the *internal* carotid can alone be endangered when the knife passes to the pharyngeal side of the styloid process, where the tumour is not likely to pass, and where it would not be safe to follow it, even were the common carotid secured, as the jugular vein and important nerves would be interfered with. These are the reasons which induce our author to think that, in such operations, it is always better to keep ourselves prepared for difficulties which possibly may occur, than, by adopting any strong measure, endeavour to anticipate those which probably shall never present themselves.

We must defer our analyses of the remaining articles in this volume till next quarter.

